# Development of a Low-Cost Passive Strain Sensor for Bridge Structural Health Monitoring

**DOI:** 10.3390/s26061963

**Published:** 2026-03-21

**Authors:** Hannah M. Power, Harry W. Shenton

**Affiliations:** 1 Mott MacDonald, Newark, DE 19716, USA; hpower@udel.edu; 2 Department of Civil, Construction, and Environmental Engineering, University of Delaware, Newark, DE 19716, USA

**Keywords:** retroreflective, bridge, tensile strain, transportation infrastructure, passive sensor, low-cost, maintenance, asset management

## Abstract

Complex structural health monitoring (SHM) systems are rarely installed on typical bridges, likely because of an expected low return on investment; however, low-cost, passive sensors made from a retroreflective sheeting material (RRSM) offer an economical alternative for SHM of typical bridges. Most departments of transportation (DOTs) fabricate and maintain traffic signs made from RRSMs. By using a material familiar to DOTs, the technology transfer from signs to strain sensing is streamlined. This paper focuses on the development of a passive strain sensor made from an RRSM. A standard Type XI fluorescent yellow-green RRSM is tested in tension to establish the relationship between retroreflectivity (RR) and induced strain. Results show RR decreases linearly with increasing strain after an initial plateau of ~1000 × 10^−6^ m/m. To function as a strain sensor, the RRSM is pre-strained beyond the plateau. A production sensor is designed to attach to the tension face of a structural element for monitoring. Periodic RR measurements are used to estimate the likely maximum strain change at the sensor location. The sensor has the potential to provide a practical, low-cost, and easily implementable solution to improve the monitoring of typical bridges, enhancing their safety and longevity.

## 1. Introduction

The U.S. Department of Transportation requires bridges to be visually inspected every 2 years. These routine inspections are qualitative in nature and yield a simple 0 to 9 condition rating for the bridge deck, superstructure, and substructure [1]. The lowest rating among the deck, superstructure, and substructure determines the overall condition of the bridge: a rating of 7 or greater is considered “good,” a rating of 5 or 6 is considered “fair,” and a rating of 4 or less is considered “poor.” The Federal Highway Administration’s (FHWA) National Bridge Inventory reports the number of good, fair, and poor condition bridges based on these inspections: as of June 2025, 43.7% were rated in good condition, 49.6% in fair condition, and 6.7% were rated in poor condition [2].

Visual inspections can vary from one inspector to another, as condition states and levels of deterioration are open to interpretation. Consequently, a bridge’s condition may be overestimated as routine inspections do not provide insight into damage that is not visible to the naked eye or is hidden beneath the surface of, for example, a reinforced concrete member. A more quantitative assessment of the bridge can be undertaken using any number of tools and methodologies. Nondestructive evaluation methodologies, such as die penetrant, chain drag, impact echo, ground penetrating radar, or ultrasonic methods, may be used to identify specific types of defects [3]. Diagnostic or proof load tests can be conducted to quantitatively evaluate the bridge’s global behavior, and the results can be used to calibrate a numerical model or develop refined load ratings for the bridge [4]. Structural health monitoring (SHM) involves installing sensors on a bridge to monitor its behavior due to live loads (traffic, wind, seismic), thermal effects, creep, shrinkage, extreme events (e.g., pier impact, crashes on or under the bridge, fire on or under the bridge, and fracture of a member), and long-term deterioration. The rates at which the data is collected in SHM can vary from a reading every few minutes, which should capture very slowly varying changes due to thermal effects, material degradation, and dead load being redistributed due to failure of a member, to tens of samples per second to capture rapidly varying changes due to live load effects. SHM systems are designed to run unattended and can be set up to operate for periods of a few weeks or months (e.g., [5,6]) or as permanent installations that are intended to operate throughout the life of the structure. A few examples of permanent SHM systems include the Indian River Inlet Bridge in Delaware [7], the Jiubao Bridge in China [8], and the Great Belt Bridge in Denmark [9].

Another tool for quantitative assessment of a bridge is a passive sensor that is only read periodically on an “as-needed” or “as-desired” basis. A key advantage of a passive sensor is that it does not require continuous power and is only read when it is energized by an external source. This type of sensor offers advantages for typical “bread-and-butter” bridges, i.e., one-, two-, and three-span highway overpasses that make up most bridges in the U.S. inventory. These bridges are unlikely to ever be monitored using sophisticated SHM systems, except in very special circumstances. As a result, there is a need to develop inexpensive, easily deployable, low-maintenance monitoring platforms for typical bridges. Passive sensors that measure strain, tilt, or deflection, which are read periodically, could provide valuable information for the long-term maintenance and operation of this class of bridges.

Passive sensors have attracted the attention of researchers in recent years. In 2013, Deivasigamani et al. [10] provided a review of research on passive sensors based on MEMS, LC/RC circuits, and antennas. This included a comparison of the advantages and disadvantages of each of these technologies [10]. Omachi et al. developed the Strain Visualization Sheet that uses the principle of Moiré fringes to passively measure strain. Strains were measured within an accuracy of +/− 50 microstrain (1 microstrain = 1 × 10^−6^ m/m = 1 µε) with the naked eye and within and less than 15 µε using image processing and photos taken with an ordinary digital camera. The sensor was also tested in field applications and showed good results [11]. Recent developments in magnetoelastic materials, such as those described by Ren et al., show that hourglass shaped magnetoelastic sensors have the potential to be more sensitive than the previously developed rectangular ones [12]. Pepakayala et al. developed a passive strain sensor that is fabricated from magnetoelastic alloys. The sensor takes advantage of the change in Young’s modulus that results from strain and magnetization of the materials [13]. The aerospace industry uses passive strain gauges for their ability to function in harsh environments where wires and batteries are prone to damage when exposed to excessive heat and loading, and sensors are often needed in areas too small to house batteries [14]. Arms et al. developed a passive peak strain sensor using a microminiature half-bridge LVDT with an entrapment collar that mechanically stores the peak strain. The sensor reliably captured the peak strain of a vibrating beam out to 3000 µε, with an error of less than 5.4% compared to a resistive strain gauge [15]. Nesser et al. reported on the development of a passive strain sensor based on an inductance–capacitance (LC) circuit with a parallel-plate capacitance sensing unit [16].

The potential for using low-cost, passive sensors for bridge monitoring is significant. One way to encourage periodic monitoring of this class of bridges is to develop low-cost, passive systems that leverage resources and technology that bridge owners are familiar with and already possess [10,17,18]. The accessibility and ease of use of a sensor like this would facilitate a quantitative analysis of bridges that otherwise would only be evaluated visually. It could be incorporated into current bridge inspection practices and used as a tool to further DOTs knowledge on how their structures are performing.

The authors have previously reported on the potential use of retroreflective sheeting material (RRSMs), which are commonly used to make highway traffic signs, as passive strain sensors. In their study, cyclic tension tests were conducted of 14 different types of RRSMs to assess the materials’ change in reflectivity with induced strain. A number of the materials exhibited sufficient strain sensitivity that they possibly could be used as a sensor for monitoring infrastructure [19]. The advantage of a RRSM based-sensing system is that bridge owners procure RRSMs to make their signs and own the instrument used to measure sign reflectivity—thus they are familiar with the materials and equipment needed to implement the sensor. This should allow for an easy transfer of the technology for bridge monitoring to owners and their consultants. Bridge owners can place RRSM sensors on their structures and periodically collect data to assess the health of the structure. The data can be collected during routine bridge inspections, allowing seamless integration of the passive strain sensing system into the existing workflow.

This paper is a follow-up to the earlier work. The novel contribution presented herein is the development and testing of a passive sensor made with a particular RRSM that can be mounted to a structural member to measure the change in strain over some period of time. This includes the design, fabrication, and testing of the prototype sensor, the development of the calibration equation, and a description of how the sensor would be used in practice. The work demonstrates a laboratory proof-of-concept of the sensor.

The paper is organized as follows: first, a description of retroreflective sheeting materials is presented, followed by results of mechanical tests conducted on an ASTM Type XI RRSM; next, the results of tension tests of the Type XI RRSM mounted to a steel dogbone specimen are presented, which is the basis for the passive sensor; this is followed by the design of the production sensor and the development of the sensor calibration equation; then, the implementation and practical application of the sensor is presented; finally, there is a discussion of how the sensor would be used in practice, followed by concluding remarks.

## 2. Retroreflective Sheeting Materials

RRSMs are flexible, reflective materials that are primarily used to fabricate traffic signs. Minimum standards for RRSMs are outlined in ASTM D4956, “Standard Specification for Retroreflective Sheeting for Traffic Control” [20]. Eleven types of material are outlined in the standard, from low-grade Type I that has the lowest reflectivity and would be used to make signs commonly found in parking lots to high-grade Type XI materials that are highly reflective and are used to make highway and construction zone signs. The proprietary materials are produced by various manufacturers and each uses unique technologies to meet the minimum standards for retroreflection [21].

**Figure 1:**
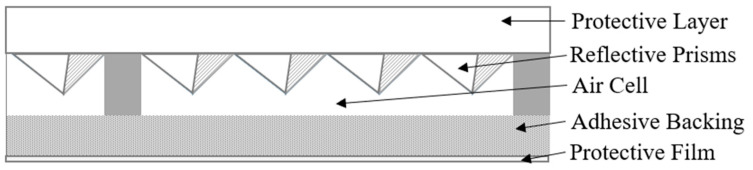
Prismatic retroreflective sheeting material cross-section.

RRSMs are composed of several basic layers: (1) a protective layer, which is exposed to the incident light and protects the reflective layer; (2) a reflective layer, which functions to reflect light and where construction varies with the ASTM type of material; (3) an adhesive layer, which, when exposed, allows the material to be adhered to a substrate; and (4) a protective film, which protects the adhesive layer until the material is ready to be adhered to a surface. Some of the lowest-grade materials use glass beads as the reflecting layer. Higher-grade materials that have higher baseline values of retroreflectivity (RR) have a prismatic reflecting layer. Figure 1 shows a schematic cross-section of a typical RRSM with a prismatic reflecting layer.

**Figure 2:**
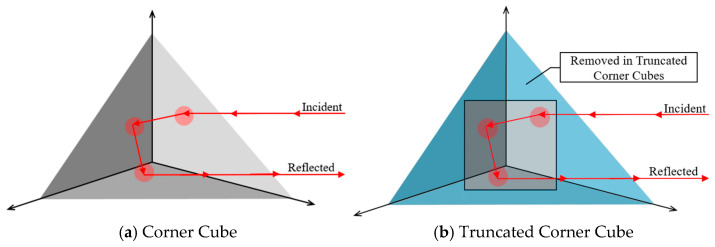
Prismatic retroreflecting prisms.

ASTM Type XI RRSMs are classified as unmetallized corner cube prismatic materials [20] and have the highest retroreflection of all RRSMs. Corner cube retroreflection works by reflecting incident light 180 degrees to another side of the prism until it is returned to the source. The space near each vertex of a corner cube prism does not reflect incident light and does not contribute to a material’s retroreflection; therefore, any light that is emitted into these areas is lost. One way to increase the efficiency of this design is to use truncated corner cube prisms, which eliminate the areas near the prism vertices that do not contribute to retroreflection. The prisms can then be stacked tightly together so that there is very little space within the reflective layer that is not contributing to retroreflection. This type of system is approximately 68% efficient [22]. Figure 2a shows a traditional corner cube prism with a retroreflected light beam, and Figure 2b highlights the areas by the prism vertices that are removed to create truncated corner cube prisms.

### 2.1. Sensor Technology Is Established in DOT Workflows

**Figure 3:**
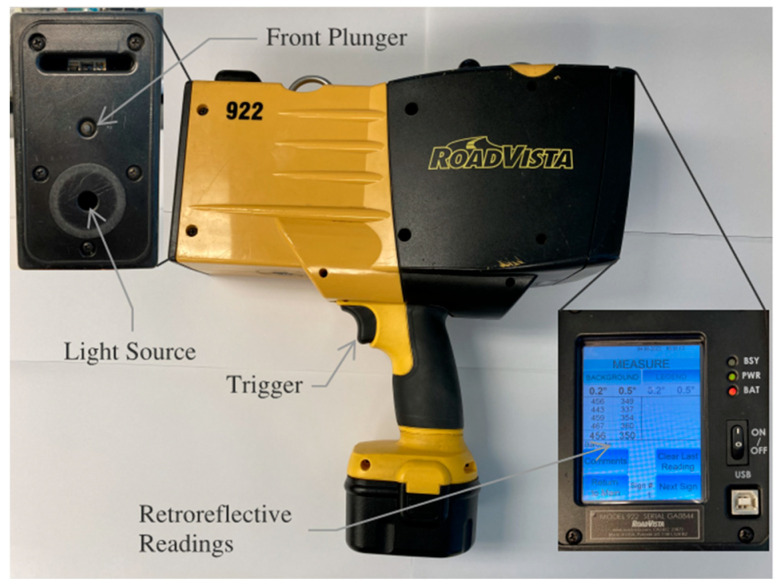
Handheld retroreflectometer.

The FHWA Manual on Uniform Traffic Control Devices [23] requires all public agencies to maintain minimum levels of sign retroreflectivity. To show compliance, agencies can measure retroreflectivity using a handheld retroreflectometer, such as the one shown in Figure 3. To make a measurement, the face of the instrument is placed flush against the RRSM, the trigger pulled, and an RR reading is displayed on the screen. The reading is a measure of the amount of light (RR) returned to the source in candelas per lux per square meter. The process is comparable to taking a temperature measurement with an infrared thermometer. ASTM minimum standards of RR for RRSMs range from a low, of about 70 for Type I materials, to a high, of about 580, for the highly reflective Type XI materials [20].

Many DOTs fabricate their own traffic signs and are required to periodically measure the retroreflectivity of their signs, thus they have access to and familiarity with retroreflectometers and RRSMs. The advantage of an RRSM passive strain sensing system is that bridge owners procure the RRSMs to make their signs and own the instrument used to measure sign reflectivity, thus they are already familiar with the materials and equipment needed to implement the strain sensor. This experience will make for an easy transfer of the technology of using RRSMs as passive strain sensors by the transportation industry.

### 2.2. Response of RRSMs to Induced Strain and Down-Selection to the Candidate RRSM

**Figure 4:**
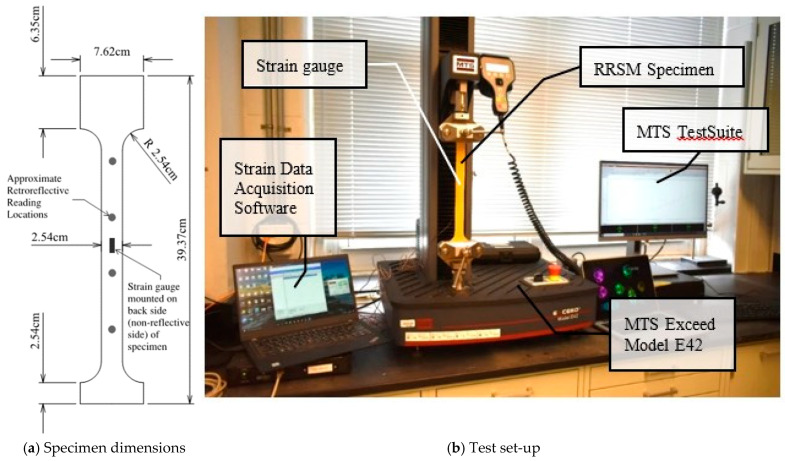
RRSM bare material tension test.

Previous work by the authors has shown that certain types of RRSM exhibit a linear relationship between induced strain (ε) and retroreflectivity—specifically, retroreflectivity decreases with increasing tensile strain [19]. Cyclic tension tests were conducted on different RRSMs to establish their sensitivity to strain. The tests were conducted on five different ASTM types (I, IV, XIII, IX, and XI) of RRSM in different colors, from two different manufacturers; a total of 14 different materials were tested. The tension tests were conducted in an MTS model E42 electromechanical test machine. The strain in the RRSM was measured using a 253SL 350-ohm resistive strain gauge manufactured by Micro-Measurements (Wendell, NC, USA) mounted to the back (non-reflective) side of the material using a quick-setting adhesive (Mbond 200) (Wendell, NC, USA). Retroreflectivity of the materials at different load/strain levels were measured using a RoadVista handheld reflectometer (Figure 3), out to a maximum strain in the material of 4000 με. The specimen and test setup are shown in Figure 4.

Many of the materials tested exhibit a linear relationship between retroreflectivity and strain, but, with varying levels of sensitivity, some exhibit a bilinear relationship. The material sensitivity to induced strain, i.e., change in retroreflectivity with strain (RR/µε), is the key factor to be determined when assessing an RRSM’s viability for use as a strain sensor. Other important factors that were assessed in the test program included: material hysteresis and degradation with loading and unloading, failure strength, and strain to failure. Based on these tests, a material was down-selected for further development into a passive sensor. That material is a Type XI (TXI-) fluorescent yellow-green (FYG) RRSM produced by one manufacturer. It is denoted here as TXIFYG.

## 3. TXIFYG Bare Material Response to Strain

TXIFYG comprises truncated corner cube prisms, and it has high sensitivity and good correlation to induced strain. For reference, the baseline (unstrained) RR of TXIFYG was measured by taking 100 retroreflective readings from a 0.09 square meter (one square foot) sample of material. The average RR was 765 cd/lx/m^2^ with a standard deviation of 40.8 cd/lx/m^2^, for a coefficient of variation of 5.3%. This is a measure of the baseline variability of the RR measurement made using a handheld retroreflectometer.

Three specimens of TXIFYG were cyclically loaded in tension while measuring the material RR and strain to determine the RR–µε relationship, as described previously. The specimens were cut into a “dogbone” shape, and a uniaxial strain gauge was mounted to the non-reflective side of the specimen (Figure 4a). The tests were conducted in an MTS Exceed E42 Electromechanical Test System (Eden Prairie, MN, USA) fitted with a 500 kgf Transcell load cell (model BSS-XS-500kg) (Buffalo Grove, IL, USA); strain was measured with a Micro-Measurements 253SL 350-ohm resistive strain gauge mounted to the back (non-reflective) side of the material using a quick setting adhesive (Mbond 200) and recorded using a Micro Measurement System 8000 data acquisition system and StrainSmart software (version 5.1) (Wendell, NC, USA) (Figure 4b). The specimen was loaded to approximately 4000 µε and unloaded three times. The first loading and unloading cycles were paused every 4.45 N (1 lb) to record strain and RR. The second and third loading and unloading cycles were paused every 8.90 N (2 lb) to record strain and RR. RRSM’s have innate variability, so four readings were taken at each load step and averaged. The average RR value was plotted versus the material strain, as measured by the resistive strain gauge to determine the RR–µε relationship.

**Figure 5:**
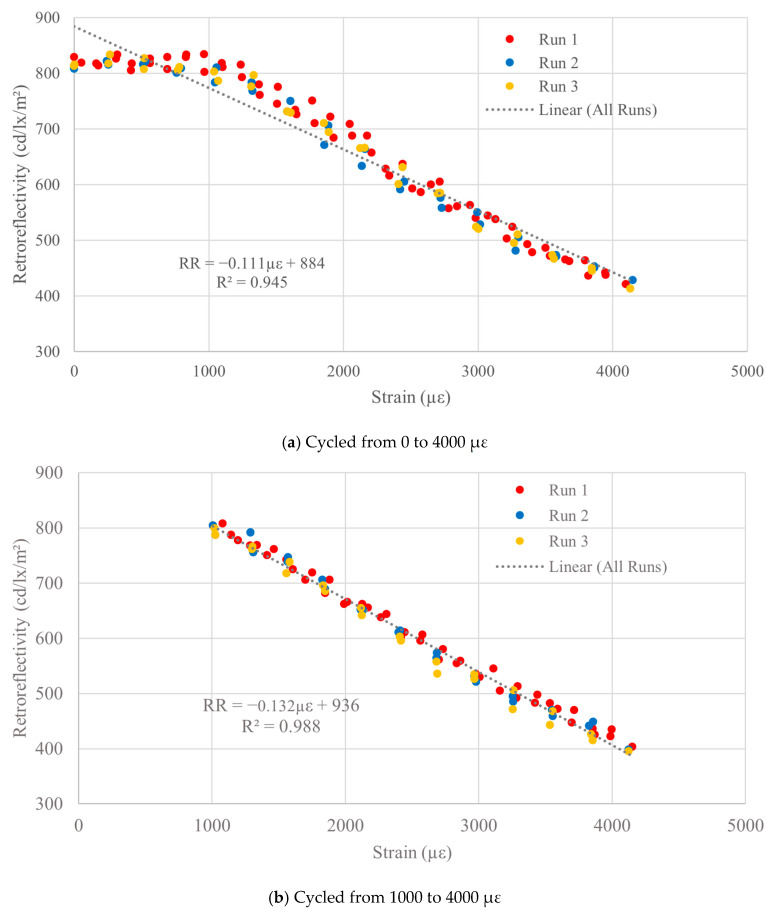
TXIFYG bare material tension test results.

Presented in Figure 5a is a plot of RR versus strain for the TXIFYG material; the plot is typical of the three replicate tests. Throughout this paper, retroreflectivity is plotted versus microstrain for the three loading and unloading cycles of a test. In this figure and similar plots, the first loading and unloading cycle data points are red, the second cycle data points are blue, and the third cycle data points are yellow. Figure 5a clearly shows RR dependence on induced strain. There is an initial nearly constant region in which the RR does not vary significantly with strain; however, after about 1000 µε, the RR decreases in a nearly linear manner with increasing strain. A linear regression line for all loading and unloading cycles from the test is plotted along with the associated equation and R^2^ value. The material sensitivity between 0 and 4000 µε is −0.111 RR/µε, with an R^2^ value of 0.949. While there is a reasonably good linear correlation over the full strain range, the data follows more of a bilinear trend with little variation in RR up to about 1000 µε.

The same specimen was tested again but first loaded to approximately 1000 µε (i.e., pre-strained) and then cycled between 1000 and 4000 µε (Figure 5b). When pre-strained and cycled between 1000 and 4000 µε, the sensitivity increased to −0.132 RR/µε, and the R^2^ increased to 0.988. Similar results were observed in the other two specimens tested. These results demonstrate that the TXIFYG material could be used to measure changes in strain by measuring changes in retroreflectivity.

## 4. Steel Mounted RRSM Response to Induced Strain

In the next phase of testing, the TXIFYG material was mounted to a steel dogbone specimen and tested in tension with the goal to confirm a similar behavior to the bare material test. However, noting the bilinear behavior of the bare material, the material was pre-strained before having been mounted to the steel dogbone so that the material would be in the linear range of response and provide a better correlation to strain over the full range of the test. Three different procedures for mounting and clamping the RRSM to the steel were tested until a satisfactory correlation was achieved. The procedures are described below; the test specimens are referred to as “unrestrained,” “gripped,” and “clamped.”

### 4.1. Preparation and Pre-Straining of the Test Specimen

**Figure 6:**
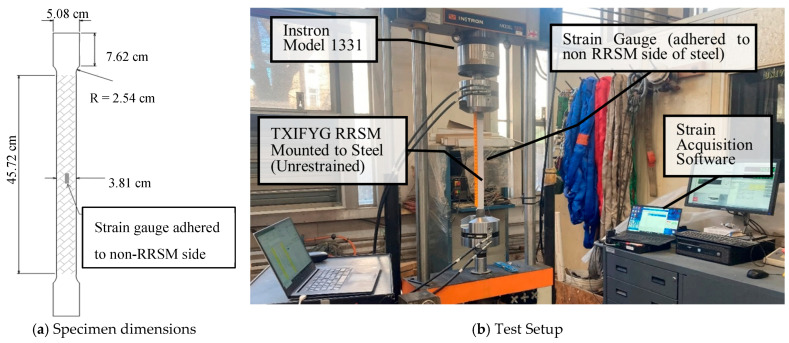
Steel-mounted TXIFYG tension test.

Strips of TXIFYG were cut, pre-strained, and mounted to 0.635 cm (1/4 inch)-thick A36 steel dogbone specimens. The design of the dogbone specimen, shown in Figure 6a, was dictated by a number of factors, including (1) the size of the aperture of the reflectometer (10 mm), (2) the need to take multiple measurements along the gauge length of the material, and (3) the capacity of the test machine. To prepare the steel surface prior to mounting the RRSM, the steel was sanded using a 220 grit sandpaper and cleaned with acetone. The TXIFYG was cut to fit the gage width and the length of the steel specimen. Pre-straining was performed by clamping the TXIFYG to a support and hanging weights from the opposite end to induce strain in the material equal to approximately 4000 µε. Retroreflective readings were taken throughout the pre-straining process to ensure the material was reaching the anticipated pre-strain. With the weights attached to the material, the material’s adhesive backing was exposed, and the TXIFYG was rolled onto the steel dogbone using a hard rubber roller. Note, the manufacturer’s adhesive was the only adhesive used to bond the TXIFYG to the steel. The simplicity in the fabrication of the RRSM sensor is intentional so that it remains low-cost and so that can be easily manufactured, installed, and read by DOTs.

### 4.2. Test Procedure

A Micro-Measurements uniaxial 253SL 350-ohm resistive strain gauge was mounted to the back of the steel dogbone specimen using a quick-setting adhesive (Mbond 200) to measure the strain in the steel. The specimens were loaded in tension in an Instron 1331 Testing Frame equipped with an Instron 200 kN load cell. The specimens were loaded to approximately 44,482 N (10,000 lb), below the yield stress of the steel, pausing every 2224 N (500 lb) to take retroreflective readings and record the strain in the steel. The specimens were then unloaded to 0 N, also pausing every 2224 N (500 lb) to take RR and strain readings. This was repeated for three loading and unloading cycles, similar to the bare material test. Sixteen RR readings were recorded along the length of the specimen; 12 readings within the center region, approximately 30 cm long, of the specimen were averaged and recorded as the RR reading for the given load. The steel specimen dimensions and test set up are shown in Figure 6.

### 4.3. Results

#### 4.3.1. Unrestrained (Figure 7a)

**Figure 7:**
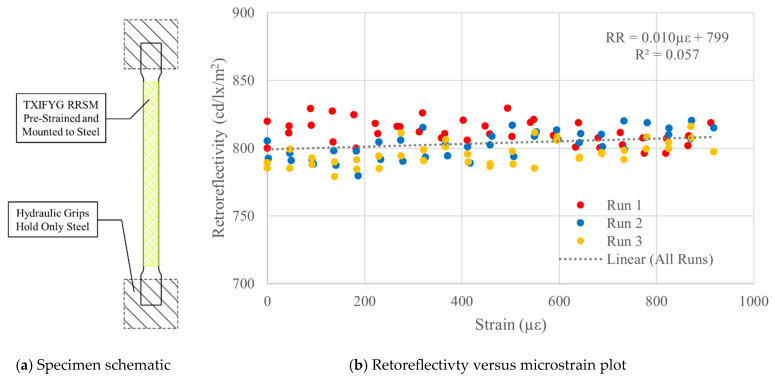
Pre-strained, steel-mounted TXIFYG on gauge length test results.

Initially, the TXIFYG was pre-strained, as described earlier, and adhered only along the gauge length of the steel specimen, i.e., the material did not extend into the grips of the test machine (Figure 7a). This was the first attempt at mounting the pre-strained RRSM to the steel substrate, and this would be the ideal scenario—that the RRSM would remain pre-strained on the steel surface without any external restraints. If this were the case, the RRSM could be adhered directly to the tension face of a structural element for passive strain monitoring.

The RR–µε relationship of this specimen did not follow that of the bare material response, as shown in Figure 7b. The data shows little correlation between RR and strain, as indicated by the very low R^2^ = 0.057. It is speculated that this was due to the large difference in the stiffness of the material relative to the steel and shear lag effects. This result demonstrated that the TXIFYG could not be used as a passive sensor by simply adhering it directly to a structural member.

#### 4.3.2. Instron Gripped (Figure 8a)

**Figure 8:**
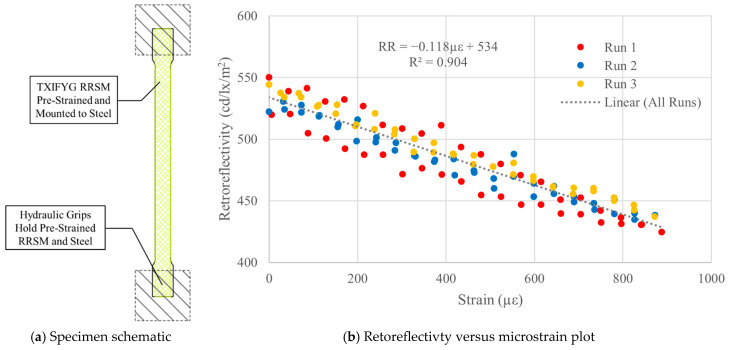
Pre-strained, steel-mounted TXIFYG in Instron grips: test results.

In the next test, the TXIFYG was pre-strained as previously described and adhered to the full length of the steel specimen so that the pre-strained material would be clamped in the test machine grips while loading (Figure 8a). This setup used external restraints (the hydraulic grips) to ensure that the pre-strain induced in the material was retained. In addition, to ensure that the RRSM remained pre-strained, when the weights were removed, it was moved quickly into the grips of the test machine. This test yielded much higher sensitivity (−0.118 RR/µε) and better linear correlation with R^2^ = 0.904 (Figure 8b).

#### 4.3.3. Externally Clamped Specimen (Figure 9a)

**Figure 9:**
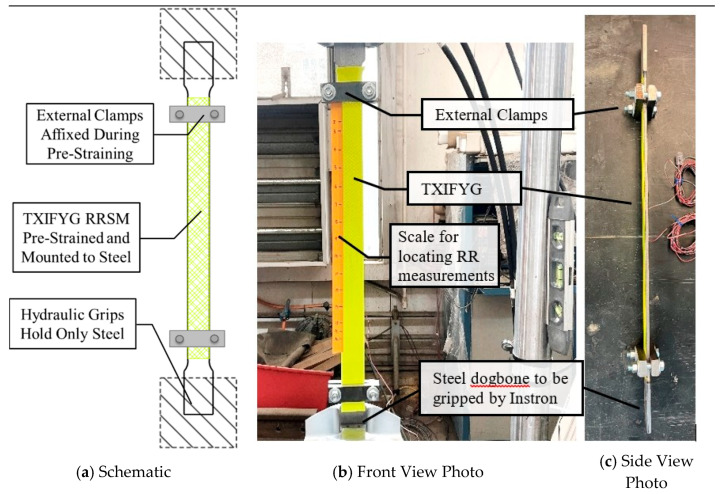
Pre-strained, clamped, steel-mounted TXIFYG specimen.

**Figure 10:**
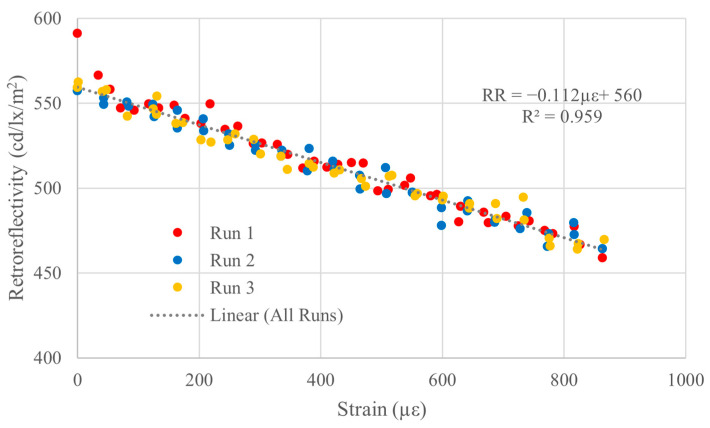
Retroreflectivity versus microstrain plot for externally clamped specimens.

The results presented in Figure 7 and Figure 8 demonstrate that for TXIFYG to be used as a passive sensor in the field, the material must be strained and then clamped to the substrate to hold the pre-strain; however, this must be done without the assistance of the test machine grips. To achieve this, a pair of steel plates were used at the top and bottom and thru-bolted to the dogbone to provide an independent clamping force to the material. The material was pre-strained as described previously, and the clamps were secured before the pre-straining weights were removed (Figure 9). Presented in Figure 10 are the test results for this specimen. This specimen’s RR–µε relationship is comparable to that of the bare material (Figure 5) and that of the gripped specimen (Figure 8b). The material sensitivity is −0.112 RR/µε with an R^2^ of 0.959 (Figure 10).

The results of the three steel mounted tests show that the TXIFYG must be externally restrained after pre-straining to achieve a well-correlated linear relationship and be viable for use as a passive strain sensor for structural health monitoring. Table 1 presents a summary of these results.

## 5. “Production” Sensor

The bare material and steel-mounted tests of the TXIFYG RRSM demonstrated its viability as a strain sensor; however, the material would need to be pre-strained and “clamped” to a surface to have a well-correlated linear strain response. Ideally, the material could be pre-strained and adhered directly to the surface on which the strain is to be measured. However, designing a method to clamp the ends of the material to the surface of a structural member would not be trivial and is counter to the objective of developing a simple, easy-to-use sensor. The alternative is to design a sensor that can hold the pre-strain, and then it would be clamped or bonded to the surface of a girder. This is similar to how other Wheatstone bridge strain transducers are utilized to conduct controlled load tests of bridges [24]. This is the approach taken to develop the production passive strain sensor.

**Figure 11:**
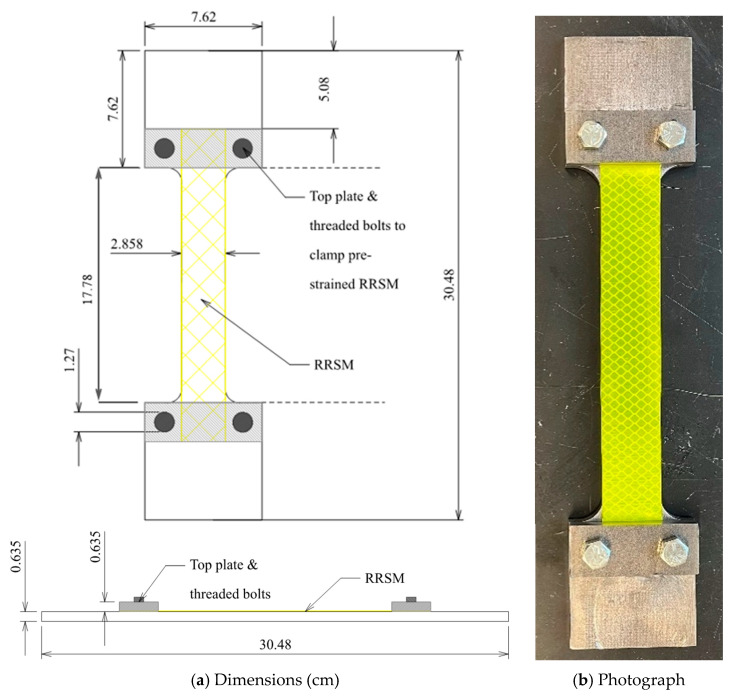
Production sensor.

The sensor shown in Figure 11 is similar to the steel dogbone specimens used in the steel mounted tests but shorter in length. It is wide enough to take RR readings with a handheld retroreflectometer, and the pre-strained RRSM is clamped using plates at each end that are bolted to the steel dogbone. With this design, the sensor can lay flush on a surface and be clamped or bonded to the structural element that is to be monitored. The gauge length is approximately 180 mm, which is slightly longer than other strain sensors used to monitor bridges today [24,25]. The ends of the sensor include flat regions where C-clamps can bear, so that it can be clamped to a surface, such as the edge of a built-up truss member or the flange of a girder.

**Figure 12:**
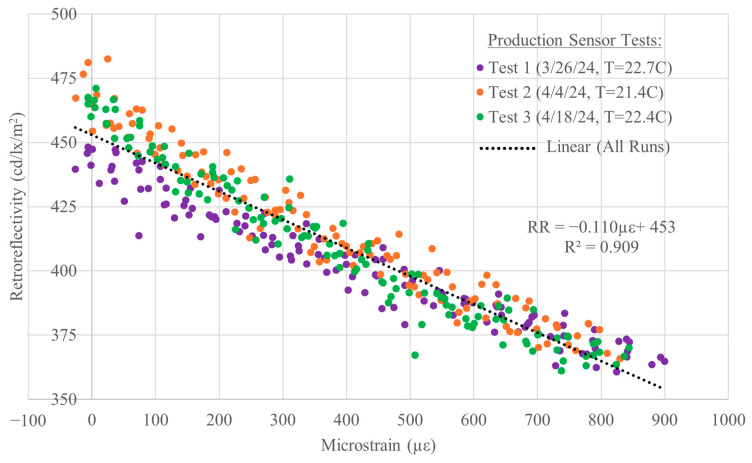
Production sensor tension test results; retroreflectivity versus microstrain with linear trendline; equation and correlation for all data.

The production sensor was subjected to the same tension test procedure described earlier (three loading and unloading cycles) on three different days (nine cycles total). The temperatures during these tests were reasonably consistent. The sensor temperature, measured using a thermocouple attached to the non-reflective side of the sensor, ranged from 21.4 °C to 22.7 °C (70.6 °F to 72.8 °F). The results of all three tests are presented in Figure 12, along with a linear best-fit line for the data from all three tests. The average RR sensitivity to strain of these three tests (−0.110 RR/µε) is comparable to the bare material results, as well as the results from the gripped specimen test (Figure 8) and from the externally clamped specimen (Figure 10). The correlation of these three tests combined is R^2^ = 0.909. These results are also presented in Table 1 for comparison to the earlier tests.

### 5.1. Calibration Equation

**Figure 13:**
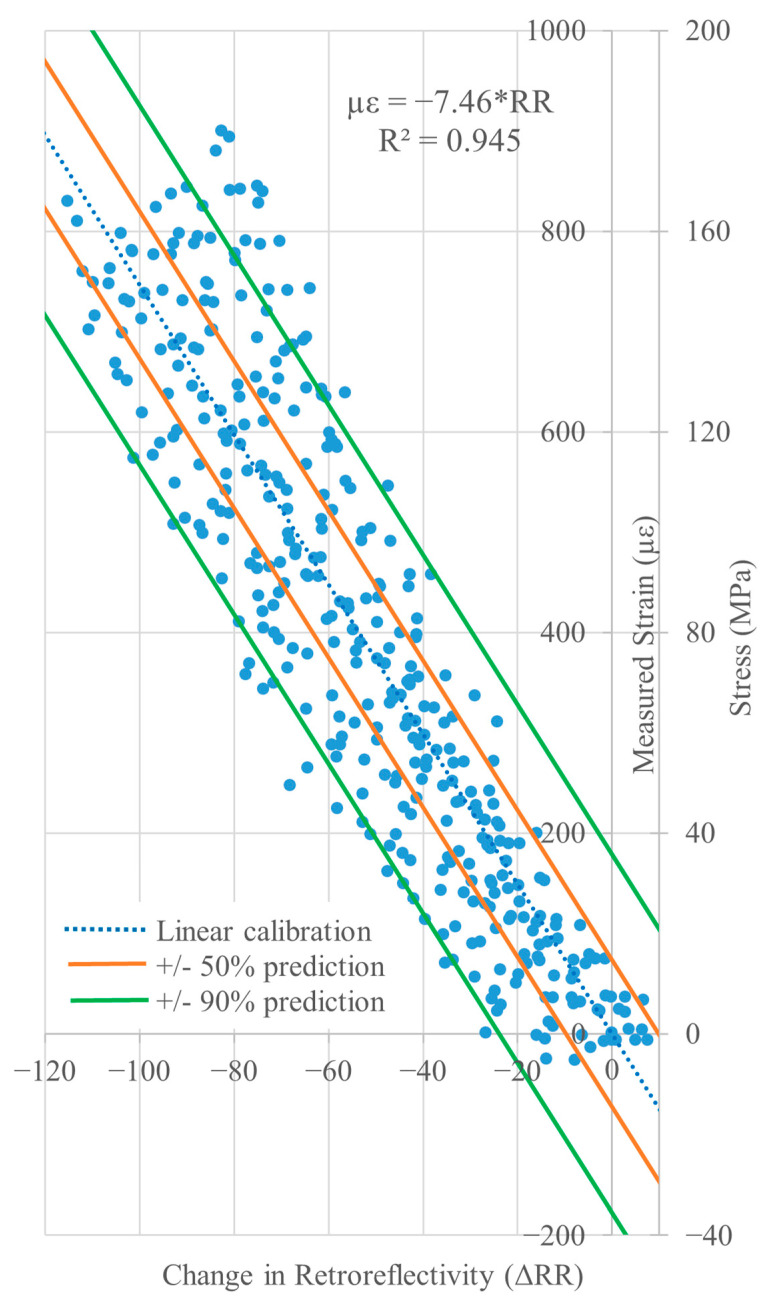
Strain versus change in retroreflectivity of production sensor with prediction interval curves (stress axis assumes E = 200 GPa).

A calibration equation for the production sensor was derived from the same data that is presented in Figure 12 by plotting strain (*y*-axis) versus retroreflectivity (*x*-axis) and subtracting the zero-strain retroreflectivity from the RR readings so that the curve starts at ~0.0. Linear regression of the data in Figure 13 yields the calibration factor for the production sensor of −7.46 με/RR; the correlation is R^2^ = 0.945.

Note, the regressions presented in Figure 12 and Figure 13 are not perfect inverses of each other because exchanging the predictor and response variables results in different predictive model parameters. In least squares regressions, the model is fit by minimizing the squared residuals in the response variable. Consequently, by interchanging the x- and y-axes, the regression in Figure 13 minimizes residuals to predict microstrain from change in retroreflectivity rather than minimizing residuals to predict RR from microstrain, as shown in Figure 12. The total sum of squares and the regression sum of squares, which are used in the calculation of R^2^, are based on the response variable; therefore, interchanging the axes results in a slightly different value for R^2^ [26].

In an actual monitoring program, the sensor would be mounted to a structural member and an initial “zero-strain” RR reading taken. This is denoted by *RR_i_* and is the value that must be subtracted from all subsequent RR readings, i.e., zeroing the initial strain reading. This is not unlike zeroing a resistive strain gauge or other sensor before starting a test to remove any initial bias in the reading. In this case, the strain measured sometime later is given by:

µε = −7.46 × Δ*RR* = −7.46 × (*RR_n_ − RR_i_*) (1)

where *RR_i_* is the initial “zero-strain” average baseline *RR* reading of the sensor at the time it was installed, and *RR_n_* is the average *RR* reading at a later time of interest; positive values indicate tensile strain.

### 5.2. Sensor Variability

Due to the variability in *RR* measurements, there is uncertainty in Equation (1) prediction. Therefore, prediction intervals were computed using JMP [27], and they are shown in orange at 50% and in green at 90% in Figure 13. These intervals estimate a range, of a given likelihood, within which an individual predicted value of microstrain is likely to fall for a given change in retroreflectivity (ΔRR). The prediction intervals can be used to determine a reasonable range of change in strain in the structure based on the RR reading [26]. Reading strain values corresponding to ΔRR from the +/−50% lines, there is a 50% chance that the actual change in strain falls between the maximum and minimum values indicated by those lines. Likewise, there is a 90% chance that the actual strain falls within the values given by the 90% lines. The “+” lines yield an upper-bound estimate of the likely maximum measured strain.

The root mean square error (RMSE) between the prediction and measured strain for this dataset is 108.2. Some of the scatter in Figure 13 can be attributed to the inherent variability of the RR measurement of the unstrained material presented earlier in Section 3.

Potential options for reducing the sensor variability are an area for continued investigation. These include: (1) modifications to the structure of the reflective material (i.e., a new design of RRSM); (2) testing of the RRSM on a more compliant substrate, such as a thinner steel dogbone, a thin aluminum dogbone, or a polymer dogbone; or (3) using duplicate sensors on the same member to provide multiple unique measurements.

### 5.3. Measurement Procedure

The following describes the procedure for using the RRSM sensor to monitor a structure.

(1) Mount the sensor to the tension face of a structural element. For example, the bottom chord or tension diagonal of a truss, a cross-bracing member, or the bottom flange of a large bridge girder.
(2) Take the initial *RR* readings along the length of the sensor and record the average value, *RR_i_*.
(3) At a later time, take new *RR* readings along the length of the sensor, record the average value *RR*_1_, and calculate Δ*RR = RR*_1_ *− RR_i_*.
(4) Calculate strain using Equation (1).
(5) Determine the desired estimated prediction interval from Figure 13 or by using the following bounds by adding to or subtracting from the calculated strain from Equation (1):
  90% prediction interval: µε ± 179.
  50% prediction interval: µε ± 73.
(6) Repeat steps #3 to #5.

## 6. RRSM Sensor in Practice

### 6.1. Example Measurement

In a hypothetical example, the RRSM sensor is used to measure the strain in a tension member of a steel truss that is the result of a heavy vehicle load being placed on the bridge. The sensor is first clamped to the member and initial readings are taken: the average reading is *RR_i_* = 500. Next, the heavy load is driven on to the bridge and parked in one location: new RR readings of the sensor are taken. The new average reading is *RR*_1_ = 460. Using Equation (1), the change in strain in the structure at the sensor location is:

µε = −7.46 × (460 − 500) = +298 µε.

For the steel bridge with E = 200 GPa, this corresponds to an increase of ~60 MPa in tension in the member (see the secondary stress axis shown in Figure 13). The 50% and 90% prediction intervals for the measurement are:

50% prediction interval: µε = 225 µε to 371 µε, in terms of stress, 45 to 74 MPa.

90% prediction interval: µε = 119 µε to 477 µε, in terms of stress, 24 to 95 MPa.

Thus, the average increase in live load stress is most likely approximately 60 MPa and not likely to be any higher than 95 MPa. The uncertainty in the result is certainly not comparable to that of a conventional strain sensor/transducer; however, the predicted strain/stress could be compared to a theoretical stress due to the load and used to assess general load distribution in the bridge: a measured value that is much greater than the theoretical one could indicate a potential problem with the bridge and trigger further analysis and testing with more sensitive, active strain sensors.

The variability of the measurement can also be assessed in relation to a typical design or yield stress. Assuming the bridge is constructed of Grade 50 steel, which has a yield stress of approximately 345 MPa (50 ksi), and assuming the design tensile strength for a member is approximately 60% of yield (207 MPa (30 ksi)), then the 50% and 90% prediction intervals represent 7% and 17% of the design stress. In the context of this example, 60 MPa corresponds to approximately 30% of the design stress. Even at the upper bound of the 90% prediction interval, 95 MPa, the stress in the member is approximately 46% of the design stress. With a calculated dead load stress, the predicted and upper-bound live load stress can be compared to the available live load capacity of the member, in effect calculating a load rating factor for the bridge. This demonstrates that, while the RRSM sensor is not as precise as conventional SHM sensors, its uncertainty still remains within limits that are practical for use in bridge health monitoring.

### 6.2. Environmental Factors

In the previous hypothetical example, because the test is conducted over a short period of time, the effects of temperature and the environment do not come into play. However, when monitoring over a period of time, these may have an influence on the sensor. Therefore, in practice, for-long term monitoring, the sensor should be protected with some manner of shield or enclosure or located on the member such that these factors are minimized to the extent possible. And, as much as possible, readings should be taken within a narrow range of temperatures. That said, work is ongoing to evaluate the effects of temperature and UV exposure on the sensor and will be reported in future publications.

### 6.3. Estimated Cost

The estimated cost of the production sensor is presented in Table 2. The material costs are very low: most of the cost is in machining the steel body and clamping plates. Assembling the sensor is very straightforward and only requires a simple hanging weight apparatus to pre-strain the TYIXFYG before it is adhered to the steel.

The total cost to fabricate a single RRSM sensor is higher than some and lower than other active conventional active sensors. For example, a single weldable resistive strain gauge costs approximately $30, while a quick-mounting full bridge strain transducer costs between $500 and $600. Including the cost of signal conditioning and data acquisition for these conventional active sensor, the cost of the passive sensor becomes more viable. This is certainly true if a DOT already owns a reflectometer to measure the reflectivity of their traffic signs.

Note that the RRSM sensor described herein is made with steel as the backing material. Other equally durable materials could be used to fabricate the sensor, such as aluminum or possibly a 3D-printed backing. It is recommended that if a material other than steel is used to fabricate an RRSM, it should be tested and calibrated.

## 7. Conclusions

Retroreflective sheeting materials, which are commonly used to make highway signs, have been shown to be sensitive to strain. This opens the door for these materials to be used for passive strain sensing of civil infrastructure systems. The TXIFYG RRSM presented in this paper has been identified as a good candidate for passive strain sensing through tension testing of the bare material and of the material mounted to a steel substrate. From this work the following key conclusions can be drawn:

1. Building upon the prior work conducted by the authors to investigate the sensitivity of RRSMs to strain, this work demonstrated a laboratory proof-of-concept of an RRSM passive strain sensor that has a range of 1000 µε.
2. A “production sensor” was designed and tested for passive monitoring of bridges. The sensor is a thin steel plate cut in the shape of a dogbone, with a Type XI RRSM adhered to one side. The size of the sensor is not unlike other strain transducers used in bridge testing that are clamped or bonded to a bridge superstructure member. The TXIFYG RRSM must be pre-strained prior to adhering to the steel and clamped at each end using restraining plates. The production sensor was tested under uniaxial tension and had a well-correlated linear relationship (R^2^ = 0.909) between RR and µε that is comparable to that of the bare TXIFYG material tension tests.
3. A sensor calibration equation was developed (Equation (1)) that can be used to calculate the change in strain at the sensor location based on changes in measured retroreflectivity.
4. The passive RRSM sensor will have variability in the calculated strain, but 50% and 90% prediction intervals were developed to estimate the likely maximum change in strain in the structure at the sensor location, with the desired level of confidence. The calculated maximum strain value is a conservative estimate of the change in strain in the structure at the sensor location. While there is high variability in the measurement, the result can still be used to compare to theoretical strain/stress predictions in the structure, which, based on the result, could trigger a detailed inspection of the structure, further analysis, and perhaps more extensive testing.
5. Using RRSMs to passively sense strain will allow for periodic structural health monitoring to be implemented on typical “bread-and-butter” bridges more regularly. Bridge owners are familiar with RRSMs and measuring their reflectivity through making and maintaining traffic signs. The sensor is inexpensive and should be within the capabilities of the DOT to fabricate. The passive RRSM sensors can be considered as another tool in the toolbox for bridge monitoring.

Work is ongoing to test the production sensor mounted to a beam loaded in bending, as well as to conduct field testing of the sensor on bridges. Work is also ongoing to investigate temperature and environmental effects on the RRSM response to strain. These results will be presented in future publications.

## Figures and Tables

**Table 1:** Summary of steel-mounted tension tests.

Test Configuration	Sensitivity (RR/µε)	R^2^
Unrestrained	0.010	0.057
Instron gripped	−0.118	0.904
Externally clamped	−0.112	0.959
Production sensor	−0.110	0.909

**Table 2:** Estimated cost of the RRSM sensor.

Item	Estimated Cost
¼” × 3” × 12” steel plate (sensor body and clamping plates)	$40
¼” hex head bolts (4 total)	$5
TYXIFYG RRSM (24” wide by 36” long sheet)	$30
Machining	$150–$300
Total	$225–$375

## Data Availability

The raw data supporting the conclusions of this article will be made available by the authors on request.

## Abbreviations

The following abbreviations are used in this manuscript:

ASTM	American Society for Testing and Materials
DOT	department of transportation
FHWA	Federal Highway Administration
SHM	structural health monitoring
RR	retroreflectivity
RRSM	retroreflective sheeting material
RMSE	root mean square error
TXIFYG	type xi fluorescent yellow-green retroreflective sheeting material
UV	ultraviolet radiation
Nomenclature	
ε	strain
µε	microstrain (10^−6^ strain)
RRi	initial retroreflective reading
RRn	retroreflective reading at time of interest
ΔRR	change in retroreflectivity
